# Effectiveness of ultrasonography and nerve conduction studies in the diagnosing of carpal tunnel syndrome: clinical trial on accuracy

**DOI:** 10.1186/s12891-018-2036-4

**Published:** 2018-04-12

**Authors:** Benedito Felipe Rabay Pimentel, Flávio Faloppa, Marcel Jun Sugawara Tamaoki, João Carlos Belloti

**Affiliations:** 1 Division of Hand Surgery and Upper Limb, Discipline of Orthopaedics and Traumatology, Paraiba Valley Regional Hospital and Taubaté University Hospital, University of Taubaté, 239 Francisco de Barros, Taubaté, São Paulo zip code 12020-230 Brazil; 2 Division of Hand Surgery and Upper Limb, Department of Orthopedics and Traumatology, Federal University of São Paulo, Paulista School of Medicine, 786 Borges Lagoa, São Paulo, São Paulo zip code 04038-030 Brazil

**Keywords:** Carpal tunnel syndrome, Diagnostic accuracy, Diagnostic practices, Clinical diagnosis, Surgical treatment, Ultrasonography, Ultrasound, Nerve conduction studies, Electrodiagnostic testing, Electromyograph

## Abstract

**Background:**

The aim of this study was to evaluate the effectiveness of two diagnostic tests routinely used for diagnosing carpal tunnel syndrome (CTS)—ultrasonography (US) and nerve conduction studies (NCS)—by comparing their accuracy based on surgical results, with the remission of paresthesia as the reference standard.

**Methods:**

We enrolled 115 patients, all of the female gender with a high probability of a clinical diagnosis of CTS. All patients underwent US and NCS for a diagnosis and subsequent surgical treatment. As a primary outcome, the accuracy of the US and NCS diagnoses was measured by comparing their diagnoses compared with those determined by the surgical outcomes. Their accuracy was secondarily evaluated based on before and after scores of the Boston Carpal Tunnel Questionnaire (BCTQ).

**Results:**

Overall, 104 patients (90.4%) were diagnosed with CTS by the surgical reference standard, 97 (84.3%) by NCS, and 90 (78.3%) by US. The concordance of NCS and surgical treatment (*p* < 0.001; kappa = 0.648) was superior to that of US and surgical treatment (*p* < 0.001; kappa = 0.423). The sensitivity and specificity of US and NCS were similar (*p* = 1.000 and *p* = 0.152, respectively: McNemar’s test). The BCTQ scores were lower after surgery in patients diagnosed by both US and NCS (*p* < 0.001and *p* < 0.001, respectively: analysis of variance).

**Conclusions:**

US and NCS effectively diagnosed CTS with good sensitivity but were not effective enough to rule out a suspicion of CTS.

**Trial registration:**

This study was registered at September, 10 th, 2015, and the registration number was NCT02553811.

**Electronic supplementary material:**

The online version of this article (10.1186/s12891-018-2036-4) contains supplementary material, which is available to authorized users.

## Background

Carpal tunnel syndrome (CTS), characterized by compression of the median nerve at the wrist level, is the most common compressive neuropathy of the upper limb [1, 2]. Ultrasonography (US) and nerve conduction studies (NCS) are diagnostic tests routinely used to confirm the diagnosis of CTS [3, 4]. As the parameters of US and NCS have not been standardization for this diagnosis, and the cutoff point for a CTS diagnosis has not been established, the diagnostic accuracy values for both tests vary widely in the literature. These variations could be related to different study designs, sample sizes, and reference standards that do not reflect routine clinical practice [1, 2]. Thus, the diagnosis of CTS is inconsistent and controversial, with no universally accepted reference standard [5, 6].

Well-designed primary CTS studies of methodological quality sufficient to guide diagnostic practices are uncommon and challenging [7–10]. Hence, we propose a strategy for diagnosing CTS in routine clinical practice. It includes a methodological resource, based on an algorithm, that could help obtain answers to the question of what is the best pathway to an accurate diagnosis of CTS. This study aimed to evaluate the effectiveness of two diagnostic tests routinely used for diagnosing CTS by comparing the accuracy of US and NCS with the results of the surgical treatment for the CTS. Remission of paresthesia following surgery was the reference standard.

## Methods

### Ethical approval, registration, guidelines and study design

The ethics and research committees of Federal University of São Paulo/Paulista School of Medicine, São Paulo, Brazil (approval No. 244468) and Paraiba Valley Regional Hospital and Taubaté University Hospital, University of Taubaté, Taubaté, Brazil (No. 009/13) approved this study. It was registered in “ClinicalTrials.gov” (No. NCT02553811) and followed the recommendations of the STARD [11]. It is a primary, longitudinal, prospective clinical trial to determine accuracy performed at a single center.

### Participants and eligibility criteria

Initially were evaluated 173 participants with a clinical suspicion of CTS. All participants who agreed to participate in this study signed the informed consent form and underwent an initial clinical evaluation by the same specialist in hand surgery. According to the sample calculation, it was necessary to include a total of 115 eligible patients.

### Probability of the clinical diagnosis: CTS-6 model

The CTS-6 [5], represents a logistic regression model that estimates the diagnostic probability of CTS. “See Additional file 1: Table S6”. A total score ≥ 12 was considered to indicate a high probability of a clinical diagnosis of CTS. There are six criteria for the probability of a clinical diagnosis (CTS-6), with their respective scores evaluated based clinical history and physical examination on the patient’s initial clinical evaluation.

1. *Paresthesia*: in the territory of distribution of the median nerve of the affected hand during any period of the day and when performing manual tasks (3.5 points)
2. *Night paresthesia*: in the territory of distribution of the median nerve of the affected hand during sleep or in the morning upon awakening (4.0 points)
3. *Weakness and hypotrophy or atrophy of the tenar musculature*: loss of strength needed to grasp objects, dropping them; evaluated by testing the tenar musculature with the thumb in opposition (5.0 points)
4. *Tinel test*: performed by applying digital percussion in the anatomical path of the median nerve at wrist level; test is positive when the patient reports a sensation of “shock” at the percussion site that irradiates to the territory of distribution of the median nerve in the affected hand (4.0 points)
5. *Phalen test*: performed by the positioning the wrist and elbow at 90° of flexion for 30–60 s; test is positive when the patient reports the onset or exacerbation of paresthesia in the territory of distribution of the median nerve of the affected hand (5.0 points)
6. *Discrimination of sensory stimuli applied between two points*: performed by applying at least 10 cutaneous sensorial stimuli using a discriminator instrument positioned longitudinally in the digital pulp of the indicators, not in direct view of the patient; test is positive when the patient does not identify at least 7 of the 10 discriminator stimuli placed at intervals of ≤6 mm (4.5 points)

### Inclusion and exclusion criteria

Inclusion criteria were a CTS-6 score of ≥12 points, female gender, aged 40–80 years, unilateral or bilateral involvement (only a more symptomatic hand was considered for inclusion), previous conservative treatment for CTS without clinical improvement. Exclusion criteria were the presence of cervical radiculopathy, thoracic outlet syndrome, pronator syndrome; a history of previous surgical release of the carpal tunnel, with sequelae of fracture of the wrist; a CTS-6 score of ≤12 points; lack of compliance at any stage of the study.

### Diagnostic interventions

After the initial physical examination, eligible patients underwent both US and NCS. These diagnostic interventions were performed in our institution, consecutively and randomly on different days by the same professional experts who were specialized and experienced with US and NCS. They were unaware of the clinical situation of the patients at the time of the examination.

### US

All US evaluations were performed on the same equipment (model M2540A; Philips Healthcare, Bothell, WA USA) with high resolution and broadband linear transducers, 4–6 MHz and 9–13 MHz. The US examination technique consisted of positioning the patient comfortably: sitting with the affected forearm in a supine position in slight flexion and supported on the examination table. The wrist is in neutral position and the fingers in extension [12]. The objective of US was to determine the cross-sectional area (CSA) of the median nerve at the proximal limit (“in let”) of the carpal tunnel by direct measurement. To evaluate the diagnostic accuracy of US, an CSA value ≥10.0 mm^2^ was the cutoff point to confirm a diagnosis of CTS [3, 12–15].

### NCS

All NCS was performed on the same equipment (model MEB 9400 K, two channels; Nihon Kohden, Tokyo, Japan). The NCS technique consisted of positioning the patient comfortably in a horizontal dorsal decubitus position with the upper limbs positioned at rest along the body and prepared for a comparative assessment of the muscular groups and the median, ulnar and radial nerves, to exclude other conditions and differential diagnoses [16–20]. The temperature of the upper limb to be examined was acclimatized at 32 °C. The age and temperature of the patients were considered in the diagnostic parameters of CTS by NCS evaluation [16]. The objective of the NCS was to determine the sensory conduction velocity (SCV) of the median nerve in the third finger–wrist segment for a length of 13 cm and the distal motor latency (DML) of the median nerve from the wrist to the tenar eminence. To evaluate the diagnostic accuracy of NCS, the cutoff values to confirm a diagnosis of CTS were < 50 m/s for SCV and ≥ 4.2 ms for DML [3, 16–20].

### Evaluation of the performance of diagnostic tests

#### Surgical treatment

After US and NCS had been performed, the patients were forwarded and underwent to surgical treatment in our institution, by one and the same surgeon. All patients underwent intravenous regional Bier block anesthesia [21]. The surgical technique used was classic open release through a palmar longitudinal incision about 2 cm in length that did not extend proximally beyond the distal flexion fold of the wrist or distally beyond the Kaplan line [22]. The patients were sent home on the same day and were followed through the fourth postoperative month [23].

#### Primary outcome

Paresthesia remission after surgical treatment was considered the reference standard for diagnosing CTS. Patients with CTS who achieved paresthesia remission postoperatively were considered truly affected by CTS, and patients who did not experience remission were considered not to have CTS [8]. The accuracy of the two preoperative diagnostics methods was measured the evaluating the US and NCS results (positive or negative) relative to the results of the surgery (remission/no remission of paresthesia) [9, 10].

#### Secondary outcome

The Boston Carpal Tunnel Questionnaire (BCTQ) is a disease-specific (CTS) questionnaire that is self-administered and has been translated into the portuguese language and validated, which evaluates two components: a scale of severity of symptoms and a scale of functional status [24, 25]. All patients in this study responded to two BCTQs: the first after the initial clinical evaluation and the second at the end of the fourth month of postoperative follow-up.

### Statistical analysis

The categorical variables were presented as relative and absolute frequencies, and numerical variables were presented as a measures summary. The evaluation of the observed and expected concordance between US and NCS and the surgical treatment were performed using the kappa coefficient. The accuracy of the US and NCS relative to the paresthesia remission after surgical treatment (reference standard) was evaluated using the McNemar. Sensitivity, specificity, positive and negative predictive values, and positive and negative likelihood ratios were analyzed by statistical calculations of expected values and obtained in a 2 × 2 contingency table. The evolution of the BCTQ score by moments of evaluation and diagnostic results was evaluated using analysis of variance with repeated measures. For all statistical tests, a significance level of 5% was adopted [26]. The statistical package SPSS 20.0 (IBM Corp., Armonk, NY, USA) and Stata12 (Stata Corp., College Station, TX, USA) software was used for the analyses.

The statistical sampling was performed considering a 20% difference between the sensitivity of the NCS and US diagnostic tests using the McNemar test, with a statistical power of 84.0% and a significance level of 5%. For this calculation, we assumed as percentage of total concordance the value of 60% and a prevalence of 80% for STC, requiring a total sample of 115 patients [26]. Statistical software PASS 14 (Power Analysis and Sample Size System; NCSS, https://www.ncss.com) was used.

## Results

### Study population

A total of 115 women were evaluated (average age ± SD 52.9 ± 9.1 years, range 40–79 years, median 52 years). The average ± SD disease duration was 4.0 ± 3.2 years, range 1–20 years, median 3 years. “See Additional file 2: Table S7”.

### Concordance between the diagnoses

Table 1 shows the total percentages of diagnoses of CTS by the US and NCS relative to the total percentage of diagnoses according to the surgical results (based on remission, or not, of paresthesia). “See Additional file 3: Table S8”.

**Table 1:** Distribution of patients by US and NCS results in relation to results of surgical treatment

Parameter	Surgical treatment				Total	
	No remission of paresthesia (CTS absent)		Remission of paresthesia (CTS present)			
	N	%	n	%	N	%
US	11	9.6	104	90.4	115	100.0
CSA < 10 mm^2^ (CTS absent)	9	7.8	16	13.9	25	21.7
CSA ≥10 mm^2^ (CTS present)	2	1.7	88	76.5	90	78.3
NCS	11	9.6	104	90.4	115	100.0
SCV ≥50 m/s and DML < 4.2 ms (CTS absent)	10	8.7	8	7.0	18	15.7
SCV < 50 m/s and DML ≥4.2 ms (CTS present)	1	0.9	96	83.5	97	84.3

*n* = 115 patients

Results are given as the total percent

*CSA* cross sectional area, *CTS* carpal tunnel syndrome, *NCS* nerve conduction studies, *DML* distal motor latency, *US* ultrasonography, *SCV* sensory conduction velocity

**Fig. 1:**
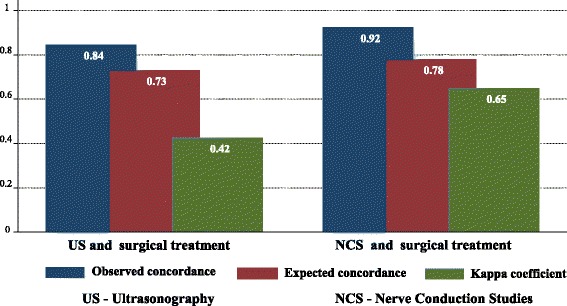
Comparison between observed, expected concordances and Kappa coefficient of the US and NCS

As seen in Table 2, there was moderate concordance between US and the surgical outcomes (*p* < 0.001, kappa = 0.423), good concordance between NCS and the surgical outcomes (p < 0.001, kappa = 0.648), and reasonable concordance between US and NCS (*p* = 0.006, kappa = 0.232) (Fig. 1).

**Table 2:** Observed and expected concordances and Kappa coefficient for the comparisons between US, NCS and surgery

Comparison	Observed concordance	Expected concordance	Kappa coefficient	Standard error	z	*p*
US vs. surgery	84.4%	72.9%	0.423	0.083	5.08	< 0.001
NCS vs. surgery	92.2%	77.8%	0.648	0.090	7.22	< 0.001
US vs. NCS	76.5%	69.4%	0.232	0.091	2.54	0.006

*NCS* nerve conduction studies, *US* ultrasonography

There were 115 subjects in each comparison group

### Accuracy of diagnostic tests

**Fig. 2:**
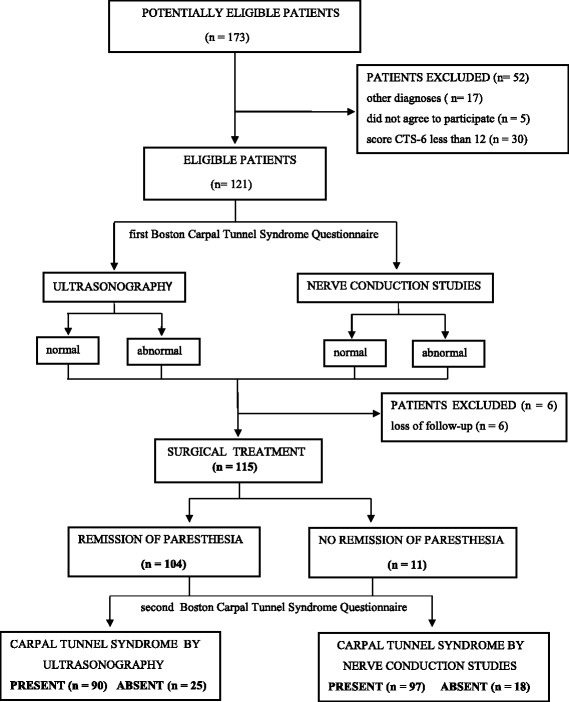
Flowchart of the diagnostic intervention results

The results of the diagnostic interventions are summarized in Fig. 2.

**Fig. 3:**
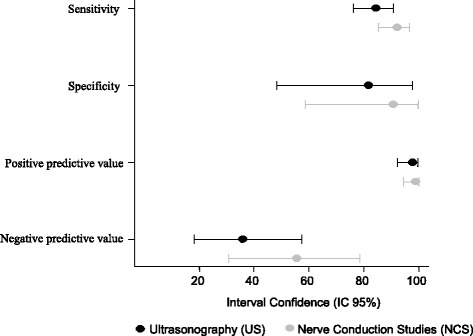
Interval confidence for sensitivity, specificity, positive and negative predictive value of the US and NCS

**Fig. 4:**
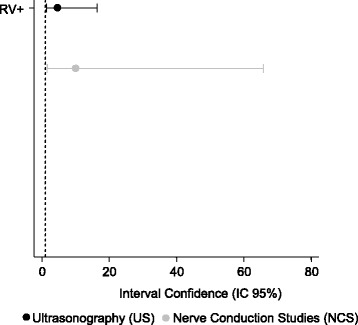
Confidence Interval for positive likelihood ratios (RV+) of the US and NCS

**Fig. 5:**
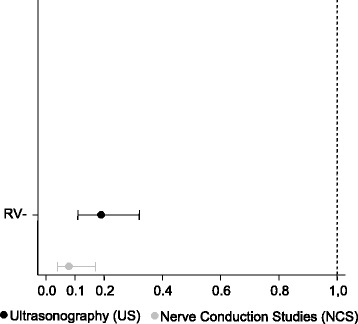
Confidence Interval for negative likelihood ratios (RV-) of the US and NCS

The sensitivity, specificity, and positive predictive values for US (*p* = 1.000, McNemar test) and NCS (*p* = 0.152, McNemar test) were similar, with no statistical differences between the two diagnostic methods (Fig. 3). The negative predictive values, however, were lower than expected for both US and NCS. “Additional file 4: Table S9, Additional file 5: Table S10, Additional file 6: Table S11, Additional file 7: Table S12”. The positive and negative likelihood values were accurate and associated with few false-positive and false-negative results for the cutoff point considered and according to the reference standard used (Figs. 4 and 5). “Additional file 8: Table S13 and Additional file 9: Table S14 respectively, shows this with more details”. The diagnostic accuracies for US and NCS, according to the statistical parameters of this study, are summarized in Table 3.

**Table 3:** Comparison of US and NCS: statistical values

Statistical parameter	US	NCS
Sensitivity(%)	84.6 (76.2–90.9)	92.3 (85.4–96.6)
Specificity(%)	81.8 (48.2–97.7)	90.9 (58.7–99.8)
Positive predictive value(%)	97.8 (92.2–99.7)	99.0 (94.4–100.0)
Negative predictive value(%)	36.0 (18.0–57.5)	55.6 (30.8–78.5)
Positive likelihood ratio(%)	4.7 (1.3–16.4)	10.2 (1.6–65.9)
Negative likelihoodratio(%)	0.2 (0.1–1.3)	0.1 (0–0.2)

*NCS* nerve conduction studies, *US* ultrasonography

### Assessment of severity of symptoms and functional status

**Fig. 6:**
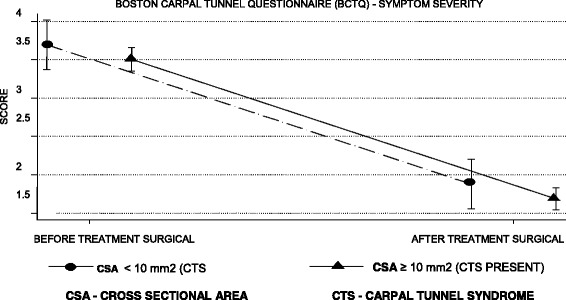
Evolution of the BCTQ score averages for symptom severity scale by the ultrasonography

**Fig. 7:**
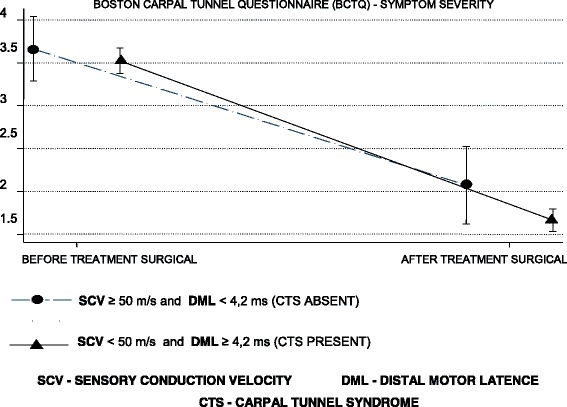
Evolution of the BCTQ score averages for symptom severity by nerve conduction studies

**Fig. 8:**
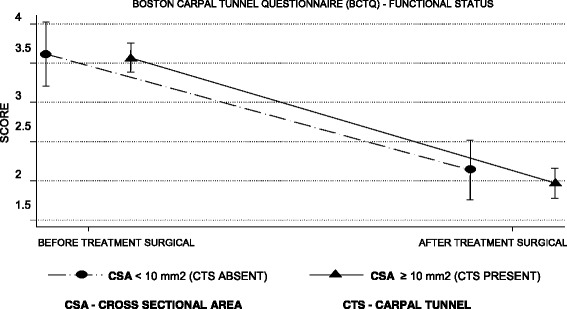
Evolution of the mean BCTQ scores for functional status by the ultrasonograph

**Fig. 9:**
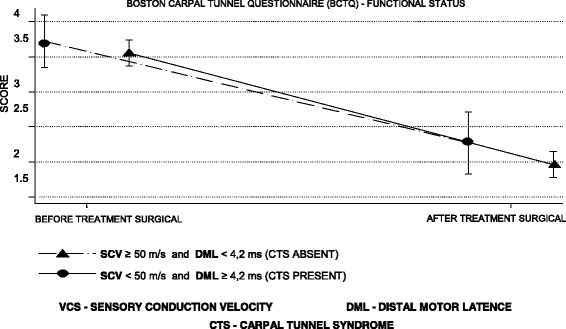
Evolution of the mean BCTQ scores for functional status by nerve conduction studies

According to Tables 4 and 5, the mean symptom severity score was reduced by 1.8 points and the functional status score by 1.6 points on the second BCTQ, applied 4 months after surgery in patients with a CTS diagnosis (Figs. 6, 7, 8 and 9).

**Table 4:** BCTQ scores for severity scale of symptoms relative to CTS diagnosis by US and NCS

Parameter	After treatment	Before treatment	Difference between after and before treatment
US			
CSA ≥10 mm^2^ (presence)	1.7 (0.7)	3.5 (0.7)	−1.8 (0.9)
CSA < 10 mm^2^ (absence)	1.9 (0.8)	3.7 (0.8)	−1.8 (1.0)
NCS			
SCV < 50 m/s and DML ≥4.2 ms (presence)	1.7 (0.7)	3.5 (0.7)	− 1.8 (0.9)
SCV ≥50 m/s and DML < 4.2 ms (absence)	2.1 (0.9)	3.7 (0.8)	−1.6 (1.0)

*n* = 115 patients

Results are given as the mean (SD)

*CSA* cross sectional area, *ANOVA* analysis of variance, *BCTQ* Boston Carpal Tunnel Questionnaire, *NCS* nerve conduction studies, *DML* distal motor latency, *US* ultrasonography, *SCV* sensory conduction velocity

ANOVA for repeated measurements—diagnostic effect: US (*p* = 0.135), NCS (*p* = 0.059)

ANOVA for repeated measures—effect of surgical treatment: US (p < 0.001), NCS (p < 0.001)

ANOVA for repeated measurements—effect of interaction between diagnosis and surgical treatment: US (*p* = 0.990), NCS (*p* = 0.246)

**Table 5:** BCTQ scores for functional status scale relative to CTS diagnosis by US and NCS

Parameter	After treatment	Before treatment	Difference between after and before treatment
US			
CSA ≥ 10 mm^2^ (presence)	2.0 (0.9)	3.6 (0.9)	−1.6 (1.1)
CSA < 10 mm^2^ (absence)	2.1 (0.9)	3.6 (1.0)	−1.5 (1.1)
NCS			
SCV < 50 m/s and DML ≥4.2 ms (presence)	2.0 (0.9)	3.6 (0.9)	−1.6 (1.1)
SCV ≥ 50 m/s and DML < 4.2 ms (absence)	2.3 (0.9)	3.7 (0.8)	−1.5 (1.1)

*n* = 115 patients

Results are given as the mean (SD)

*CSA* cross sectional area, *ANOVA* analysis of variance, *BCTQ* Boston Carpal Tunnel Questionnaire, *NCS* nerve conduction studies, *DML* distal motor latency, *US* ultrasonography, *SCV* sensory conduction velocity

ANOVA for repeated measurements—diagnostic effect: US (*p* = 0.634), NCS (*p* = 0.821)

ANOVA for repeated measures—effect of surgical treatment: US (*p* < 0.001), NCS (*p* < 0.001)

ANOVA for repeated measures—effect of interaction between diagnosis and surgical treatment: US (*p* = 0.629), NCS (*p* = 0.622)

## Discussion

Well-designed studies that have focused on the diagnosis of CTS are uncommon. The deficiencies in the design of studies involving clinical and complementary tests for CTS are associated with a super-estimation of the performance of these diagnostic tests and the lack of a universally accepted reference standard for diagnosing CTS [6, 8–10]. Most CTS diagnostic accuracy studies were unlikely to report results that are applicable in clinical practice [1, 27]. The current study design followed an algorithm based on evaluating an actual clinical practice routine for diagnosing CTS: from the consecutive and random eligibility of patients based on a high probability of a clinical diagnosis of CTS, confirmed (or not) by US and NCS—performed such that the examiners were unaware of the clinical condition of the patients and the results of the concurrent complementary examination treatment follow-up and its clinical outcomes. To ensure good diagnostic accuracy it is important that the estimates provided by a diagnostic test reflect its good performance in clinical practice [1]. The remission of paresthesia (reference standard) after surgical treatment was used in this study to evaluate the effectiveness and performance of the diagnosis of CTS by the US and NCS [3]. The classical open approach is considered a definitive surgical treatment for CTS with good results in 75 to 90% of patients [23]. In this study, 90.4% of the 115 operated patients obtained remission of paresthesia with four months pos operative, while in another study 78.9% of the 113 operated patients obtained remission of paresthesia with ten years pos operative by same surgical technique. [28]. As for the 9.6% of operated patients who did not obtain remission of paresthesia with surgical treatment in our study, it can be attributed to proximal compressions of the median nerve, double cervical compression syndrome, cervical radiculopathy or even to an advanced degree of CTS [22]. The evaluation of the patients who did not obtain remission of paresthesia after 4 months of the surgical treatment was based on clinical criteria, using the parameters to the CTS-6 and secondly BCTQ [1, 5, 24]. Though the false-negative patients had normal (absent) results for STC by US and NCS in this study, they were submitted to surgical treatment, supported by the results obtained by the clinical diagnosis for CTS in the initial clinical evaluation. Patients with normal results (absent) for CTS by the US showed better performance in the remission of paresthesia after surgical treatment, as shown in the Table 1. To obtain good quality of evidence in this accuracy study, the degree of CTS impairment provided by US and NCS was discarded and reduced to a simple dichotomous (present / absent, abnormal / normal) diagnosis to make these tests useful in clinical practice of routine [8, 9, 11]. The parameters and cut-off points considered in this study for the diagnosis of CTS by the US and NCS had a direct impact on the primary and secondary outcomes, producing the four possible types of results of a diagnostic test, showing balanced results for the values of accuracy, especially between sensitivity and specificity. The positive results obtained by the kappa index in the evaluation of NCS and US concordance in relation to the reference standard in this study expressed the reliability of these tests for the diagnosis of CTS, with NCS showing a better performance. Mondelli et al. evaluated the diagnostic usefulness of US and NCS in patients with a clinical diagnosis of CTS and obtained positive kappa values with good concordance [19]. The confirmation of the clinical diagnosis of CTS in 90.4% of patients, with a total of 84.3% for NCS and 78.3% for the US—using remission of paresthesia as the reference standard—validated the CTS-6 clinical diagnostic probability instrument used during the initial clinical evaluation. Fowler et al. compared US and NCS using CTS-6 as a reference standard and obtained results relative to confirmation of the clinical diagnosis of CTS in 65% of patients, relative to US diagnosis in 61%, and relative to NCS diagnosis in 65% [29].

The sensitivity, specificity, and positive predictive value of the US and NCS obtained in the current study were similar, showing that both diagnostic methods were effective for diagnosing CTS, with good sensitivity. Well-designed diagnostic accuracy studies with STARD-compliant methodology obtained similar results when comparing US and NCS [15, 29, 30]. Because of the low negative predictive values, however, neither US nor NCS could adequately rule out the clinical suspicion of CTS in this study when compared with other studies with different reference standards [3, 29–32].

The reduced average of the two scores obtained with the second BCTQ were statistically significant in the current study, indicating improvement of the paresthesia. Thus, the results of the surgical treatment in this study were effective, according to the minimal clinical difference proposed by Ozer et al. [33].

The main limitation of this study was that we did not consider the degree of severity of the initial clinical and complementary diagnosis of CTS. The two-point discrimination test and atrophy of the thenar musculature used in the CTS-6 model are directly linked to the severity of CTS [34]. A value of > 13 mm2 for the median nerve area found during US would correspond to a moderate degree of impairment [35, 36]. Absence of a sensory response and abnormal distal motor latency during NCS would correspond to a severe degree of impairment [3, 19].

Another limitation of this study was the inclusion of only female patients, having the disadvantage of limiting the generalization capacity of available information [10].

## Conclusions

US and NCS were effective in the diagnosis of CTS, showing a good sensitivity for the detection of patients with CTS. The US and NCS did not show adequate complementary exams to avoid the clinical suspicion of CTS. The NCS presented better concordance regarding the reference standard (remission of paresthesia) than the US. The CTS-6 clinical diagnostic probability instrument was effective and validated for the suspected cases of CTS, through the results obtained by the reduction of the means of the scores at the postoperative in relation to the preoperative evaluated by the BCTQ. The conclusions obtained by study will be useful for areas that routinely handle the diagnosis-treatment algorithm of CTS such as rheumatology, neurology, neurosurgery and orthopedics. This accuracy clinical trial provided further data regarding clinical diagnosis and surgical treatment that may be useful in future research.

## Additional files

Additional file 1: **Table S6.** Clinical diagnostic probability instrument CTS-6. (DOCX 17 kb)

Additional file 2: **Table S7.** Distribution of patients by clinical characteristics. (DOCX 17 kb)

Additional file 3: **Table S8.** Distribution of patients in percentages of CTS diagnosis for US, NCS and reference standard. (DOCX 14 kb)

Additional file 4: **Table S9.** Sensitivity and specificity of the US in relation to the reference standard. (DOCX 14 kb)

Additional file 5: **Table S10.** Sensitivity and Specificity of the NCS in relation to the reference standard. (DOCX 14 kb)

Additional file 6: **Table S11.** Positive and negative predictive value of the US in relation to the reference standard. (DOCX 14 kb)

Additional file 7: **Table S12.** Positive and negative predictive value of the NCS in relation to the reference standard. (DOCX 14 kb)

Additional file 8: **Table S13.** Positive and negative likelihood ratios of US in relation to the reference standard. (DOCX 14 kb)

Additional file 9: **Table S14.** Positive and negative likelihood ratios of the NCS in relation to the reference standard. (DOCX 14 kb)

## Abbreviations

BCTQ: Boston carpal tunnel questionnaire
CSA: Cross sectional area
CTS: Carpal tunnel syndrome
CTS-6: Instrument of probability diagnostic for carpal tunnel syndrome
DML: Distal motor latency
NCS: Nerve conduction studies
SCV: Sensory conduction velocity
STARD: Standards for reporting of diagnostic accuracy studies
US: Ultrasonography
