# Supplementary material for: Effectiveness of ultrasonography and nerve conduction studies in the diagnosing of carpal tunnel syndrome: clinical trial on accuracy
Source: BMC Musculoskelet Disord. 2018 Apr 12;19:115. doi: 10.1186/s12891-018-2036-4 (PMC5898048; doi:10.1186/s12891-018-2036-4)
Supplement: Supplementary file 1 — Table S6. Clinical diagnostic probability instrument CTS-6. (DOCX 17 kb) [file 12891_2018_2036_MOESM1_ESM.docx]

**Table S6.** Clinical diagnostic probability instrument CTS-6

| CRITERIA FOR CLINICAL DIAGNOSIS OF CTS | DIAGNOSTIC EVALUATION | INCLUSION CRITERIA | POINTS |
| --- | --- | --- | --- |
| 1 – PARESTHESIA (median nerve distribution territory) | Clinical history | Present (+) | 3,5 |
| 2 – NIGHT PARESTHESIA | Clinical history | Present (+) | 4,0 |
| 3 – WEAKNESS AND  HYPOTROPHY OR THENAR MUSCLE ATROPHY | Clinical history    Physical examination: inspection of the thenar muscles thumb. | Present (+)    Test + presence of hypotrophy or atrophy. | 5,0 |
| 4 – TINEL SIGN | Physical examination: digital percussion in the anatomical path of the median nerve at the wrist. | Test + choking sensation at the site of percussion and irradiation to the median nerve distribution territory. | 4,0 |
| 5 – PHALEN TEST | Physical examination: positioning of the wrist and elbow at 90° of flexion for 60 seconds | Test + feeling of discomfort and paresthesia in the median nerve distribution territory. | 5,0 |
| 6 – LOSS OF TWO-POINT DISCRIMINATION | Physical examination: 10 skin patch stimuli through a discriminator instrument at the fingertip of the indicator, without direct vision of the patient. | Test + patient does not identify at least 7 stimuli with discriminator instrument at a distance of ≥6 mm. | 4,5 |

CTS-6: validated clinical diagnostic tool for carpal tunnel syndrome according to Graham et al^5^.
