# Supplementary material for: Effectiveness of ultrasonography and nerve conduction studies in the diagnosing of carpal tunnel syndrome: clinical trial on accuracy
Source: BMC Musculoskelet Disord. 2018 Apr 12;19:115. doi: 10.1186/s12891-018-2036-4 (PMC5898048; doi:10.1186/s12891-018-2036-4)
Supplement: Supplementary file 2 — Table S7. Distribution of patients by clinical characteristics. (DOCX 17 kb) [file 12891_2018_2036_MOESM2_ESM.docx]

**Table S7.** Distribution of patients by clinical characteristics

| **Clinical characteristics** | **N** | **%** |
| --- | --- | --- |
| **Dominant hand** | **115** | **100,0%** |
| right | 113 | 98,3% |
| left | 2 | 1,7% |
|  |  |  |
| **Involvement** | **115** | **100,0%** |
| bilateral | 109 | 94,8% |
| unilateral | 6 | 5,2% |
|  |  |  |
| **Side affected** | **115** | **100,0%** |
| right | 59 | 51,3% |
| left | 56 | 48,7% |
|  |  |  |
| **Diabetes melitus** | **115** | **100,0%** |
| no | 97 | 84,3% |
| yes | 18 | 15,7% |
|  |  |  |
| **Systemic arterial hypertension** | **115** | **100,0%** |
| no | 70 | 60,9% |
| yes | 45 | 39,1% |
|  |  |  |
| **Hypothyroidism** | **115** | **100,0%** |
| no | 108 | 93,9% |
| yes | 7 | 6,1% |
|  |  |  |
| **Renal insufficiency** | **115** | **100,0%** |
| no | 113 | 98,3% |
| yes | 2 | 1,7% |
|  |  |  |
| **Rheumatopathies** | **115** | **100,0%** |
| no | 109 | 94,8% |
| yes | 6 | 5,2% |
|  |  |  |
| **Previous clinical treatment** | **115** | **100,0%** |
| yes | 115 | 100,0% |

n=115 patients.

Results are given as the total percent.
