# Supplementary material for: Effectiveness of ultrasonography and nerve conduction studies in the diagnosing of carpal tunnel syndrome: clinical trial on accuracy
Source: BMC Musculoskelet Disord. 2018 Apr 12;19:115. doi: 10.1186/s12891-018-2036-4 (PMC5898048; doi:10.1186/s12891-018-2036-4)
Supplement: Supplementary file 3 — Table S8. Distribution of patients in percentages of CTS diagnosis for US, NCS and reference standard. (DOCX 14 kb) [file 12891_2018_2036_MOESM3_ESM.docx]

**Table S8.** Distribution of patients in percentages of STC diagnosis for US, NCS and reference

standard

| **CTS diagnosis** | **Evaluation** | | | | | |
| --- | --- | --- | --- | --- | --- | --- |
|  | **US** | | **NCS** | | **Reference standard** | |
|  | **n** | **%** | **n** | **%** | **N** | **%** |
| Presence | 90 | 78,3 | 97 | 84,3 | 104 | 90,4 |
| Absence | 25 | 21,7 | 18 | 15,7 | 11 | 9,6 |

n=115 patients.

Results are given as the total percent.

CTS, carpal tunnel syndrome; US, ultrasonograph; NCS, nerve conduction studies.
