# Supplementary material for: Effectiveness of ultrasonography and nerve conduction studies in the diagnosing of carpal tunnel syndrome: clinical trial on accuracy
Source: BMC Musculoskelet Disord. 2018 Apr 12;19:115. doi: 10.1186/s12891-018-2036-4 (PMC5898048; doi:10.1186/s12891-018-2036-4)
Supplement: Supplementary file 6 — Table S11. Positive and negative predictive value of the US in relation to the reference standard. (DOCX 14 kb) [file 12891_2018_2036_MOESM6_ESM.docx]

**Table S11.** Positive and negative predictive value of the US in relation to the reference standard

|  | Surgical treatment | | | | Total | |
| --- | --- | --- | --- | --- | --- | --- |
|  | Remission of paresthesia  (Presence) | | No remission of paresthesia (Absence) | |  |  |
|  | N | % | N | % | N | % |
| US | 104 | 90.4% | 11 | 9.6% | 115 | 100.0% |
| CSA ≥ 10 mm2 (Presence) | 88 | **97.8%*** | 2 | 2.2% | 90 | 100.0% |
| CSA < 10 mm2 (Absence) | 16 | 64.0% | 9 | **36.0%†** | 25 | 100.0% |

*** Positive predictive value; † negative predictive value.**

n=115 patients.

Results are given as the total percent.

US, ultrasonograph; CSA, cross sectional area.
