# Supplementary material for: Effectiveness of ultrasonography and nerve conduction studies in the diagnosing of carpal tunnel syndrome: clinical trial on accuracy
Source: BMC Musculoskelet Disord. 2018 Apr 12;19:115. doi: 10.1186/s12891-018-2036-4 (PMC5898048; doi:10.1186/s12891-018-2036-4)
Supplement: Supplementary file 8 — Table S13. Positive and negative likelihood ratios of US in relation to the reference standard. (DOCX 14 kb) [file 12891_2018_2036_MOESM8_ESM.docx]

**Table S13.** Positive and negative likelihood ratios of US in relation to the reference standard

|  | Surgical treatment | | | | Total | |
| --- | --- | --- | --- | --- | --- | --- |
|  | Remission of paresthesia  (Presence) | | No remission of paresthesia  (Absence) | |  |  |
|  | N | % | N | % | N | % |
| US | 104 | 100.0% | 11 | 100.0% | 115 | 100.0% |
| CSA ≥ 10 mm2 (Presence) | 88 | 84.6% | 2 | 18.2% | 90 | 78.3% |
| CSA < 10 mm2 (Absence) | 16 | 15.4% | 9 | 81.8% | 25 | 21.7% |

**Positive likelihood ratios = 4.7; negative likelihood ratios = 0.2**

n=115 patients.

Results are given as the total percent.

US, ultrasonograph; CSA, cross sectional area.
