# Supplementary material for: Effectiveness of ultrasonography and nerve conduction studies in the diagnosing of carpal tunnel syndrome: clinical trial on accuracy
Source: BMC Musculoskelet Disord. 2018 Apr 12;19:115. doi: 10.1186/s12891-018-2036-4 (PMC5898048; doi:10.1186/s12891-018-2036-4)
Supplement: Supplementary file 9 — Table S14. Positive and negative likelihood ratios of the NCS in relation to the reference standard. (DOCX 14 kb) [file 12891_2018_2036_MOESM9_ESM.docx]

**Table S14.** Positive and negative likelihood ratios of the NCS in relation to the reference standard

|  | Surgical treatment | | | | Total | |
| --- | --- | --- | --- | --- | --- | --- |
|  | Remission of paresthesia  (Presence) | | No remission  of paresthesia  (Absence) | |  |  |
|  | N | % | N | % | N | % |
| NCS | 104 | 100.0% | 11 | 100.0% | 115 | 100.0% |
| SCV < 50 m/s and DML ≥ 4,2 ms  (Presence) | 96 | 92.3% | 1 | 9.1% | 97 | 84.3% |
| SCV ≥ 50 m/s and DML < 4,2 ms  (Absence) | 8 | 7.7% | 10 | 90.9% | 18 | 15.7% |

**Positive likelihood ratios = 10.2; negative likelihood ratios = 0.1**

n=115 patients.

Results are given as the total percent.

NCS, nerve conduction studies; SCV, sensory conduction velocity; DML, distal motor latency.
